# Quantitative eDNA abundances for multispecies monitoring along the US West Coast

**DOI:** 10.7717/peerj.21810

**Published:** 2026-11-09

**Authors:** Gledis Guri, Owen R. Liu, Ryan P. Kelly, Megan R. Shaffer, Kim M. Parsons, Ana Ramón-Laca, Krista M. Nichols, Pedro F. P. Brandao-Dias, Abigail Wells, Andrew O. Shelton

**Affiliations:** 1 School of Marine and Environmental Affairs, University of Washington, Seattle, Washington, United States; 2 Conservation Biology Division, Northwest Fisheries Science Center, NOAA Fisheries, Seattle, Washington, United States; 3 School of Aquatic and Fishery Sciences, University of Washington, Seattle, Washington, United States; 4 Museo Nacional de Ciencias Naturales CSIC, Madrid, Spain

**Keywords:** Bio-monitoring, eDNA, Fish, Natural resources, Quantification, Pacific, Coastal pelagic species

## Abstract

Molecular data from environmental samples can reflect the abundance of species’ DNA, an index immediately relevant to natural-resources management at a broad geographic scale. These data commonly derive from assays designed and targeted for individual species (*e.g*., using quantitative polymerase chain reaction (qPCR) or digital droplet polymerase chain reaction (ddPCR)), or from metabarcoding that use more general PCR primers to amplify many species simultaneously. Multispecies analyses make efficient use of field samples and laboratory time and speak to inherently multispecies questions of management and ecological interest. However, unlike single-species techniques, metabarcoding alone generally reflects only the environmental DNA (eDNA) proportions of target-species DNA present, not their absolute quantities. Here, we combine qPCR and metabarcoding data derived from the same samples to estimate quantities of eDNA from many fish species along the US West Coast in three dimensions, demonstrating a technique of practical relevance for both management and ecology. We derive spatially explicit maps of eDNA abundances for 12 common species of ecological and management importance and point the way to quantitative surveys of wild species using molecules alone. We find that species distribution maps derived from eDNA largely mirror known species niches and known spatial distributions. Notably, our analyses identify biodiversity hotspots that align with previously documented regions of ecological significance, such as the Columbia River plume and the Heceta Bank.

## Introduction

Surveys of marine environments play a fundamental role in supporting fisheries and conservation of protected species but are often difficult and expensive endeavors. Although trawls, acoustic surveys, and other traditional sampling methods have long been used to directly measure species abundance and biomass, they can be impractical for species inhabiting inaccessible habitats, occurring at low densities, or species that are easily injured during capture.

Environmental DNA (eDNA; genetic material sampled from water, soil, or air) is an increasingly common tool for noninvasively sampling marine and aquatic ecological communities, valuable for estimating species occurrence (Veilleux, Misutka & Glover, 2021), biodiversity (*i.e*., richness; Muenzel et al., 2024), and genetic structure (Andres, Lodge & Andrés, 2023). eDNA sampling captures information for a broad range of taxa, including those that are difficult or impossible to study with traditional gear (Ledger et al., 2025). As such, eDNA can meaningfully complement traditional survey methods for commercially important species while at the same time revealing the abundance and distribution of elusive or understudied species (Johnson et al., 2025).

However, many practical management and conservation applications rely on quantifying the abundance of target species, rather than its residual traces. This is an area of much interest and developing techniques in the context of eDNA studies (see Shelton et al., 2022; Stoeckle, Ausubel & Coogan, 2022; Guri et al., 2024). The use of eDNA for quantification is supported both conceptually—where there is more of a species, more cells are shed in the environment, and thus we expect more DNA. Strong positive relationships between eDNA concentration and abundance and/or biomass have been found in mesocosm experiments (Takahara et al., 2012; Doi et al., 2015; Karlsson et al., 2022; Ledger et al., 2024) and in field studies (see review by Rourke et al. (2022)). In field settings, strong eDNA-abundance/biomass relationships have been found across many survey methods, including electrofishing (Baldigo et al., 2017; Jacobsen et al., 2023), acoustics (Jo et al., 2017), trap catches (Nevers et al., 2018), visual census and mark-recapture (Yamamoto et al., 2016; Levi et al., 2019; Kirtane et al., 2021), and trawl-based estimates (in rivers (Pont, 2024), estuaries (DiBattista et al., 2022; Baetscher, Nuetzel & Garza, 2023), and coastal seas (Maes et al., 2023)).

Nevertheless, a range of physical, biological, and analytical processes intervenes between organismal abundance and observed eDNA concentration (Collins et al., 2018; Guri et al., 2024; Xiong et al., 2025), such that no one link function is likely to capture this relationship across species or environmental contexts. As a result, real-world quantitative applications can take years of species-specific analysis. For example, for the first time in 2025 the U.S.-Canada joint Pacific hake stock assessment *(Merluccius productus)* made use of an eDNA-derived abundance index for the species under management (Johnson et al., 2025), building upon (Shelton et al., 2022) to use molecular observations in a formal process alongside more traditional acoustic and trawl surveys in a fisheries assessment.

However, single species approaches remain impractical for large-scale applications because each species requires independent assay development, optimization, and processing (Langlois et al., 2021; Ruppert et al., 2022). In contrast, multispecies analysis (metabarcoding) reflects information on many species of interest simultaneously, but at the costs of producing compositional estimates of proportional abundance (Gloor et al., 2017; McLaren, Willis & Callahan, 2019) that may require statistical correction for species-specific biases (Shelton et al., 2023), shaped in part by polymerase chain reaction (PCR) amplification but potentially also by other factors such as differential shedding rates linked to spawning events, lifestyle, or body physiology (Guri et al., 2024). Although a few studies have calibrated metabarcoding results against quantitative or independent abundance measures, these efforts have so far been confined to small regional scales (Stoeckle, Ausubel & Coogan, 2022; Guri et al., 2024), highlighting the need for methods that extend such approaches to broader, management-relevant spatial domains.

The first step toward scaling up molecular monitoring for regulatory and management applications—which require abundance estimates of the species being managed—is quantifying eDNA concentrations for many species over a large geographic area under management, such as we present here. We develop quantitative estimates of eDNA abundance for a diverse group of fish species spanning different depth niches across 10 degrees of latitude along the US West Coast entirely from eDNA. We integrate single and multi-species (using MiFish-U universal primers (Miya et al., 2015)) techniques to create three-dimensional maps of eDNA concentration (Fig. 1), leveraging models established in previous studies (Shelton et al., 2022, 2023; Allan et al., 2023; Guri et al., 2024). Rather than advancing a new methodological framework, this study tests whether multi-species eDNA absolute abundance derived from metabarcoding captures known species distributions and depth habitat niches at large scales—a spatial domain for which direct point-by-point validation against trawl or acoustic surveys is not operationally feasible. Although here we quantify eDNA concentration—and we emphasize, not biomass—for our target set of fish species, our results suggest that the smoothed spatial distribution of eDNA concentration is likely in many cases to reflect the distribution of the fish species themselves.

**Figure 1:**
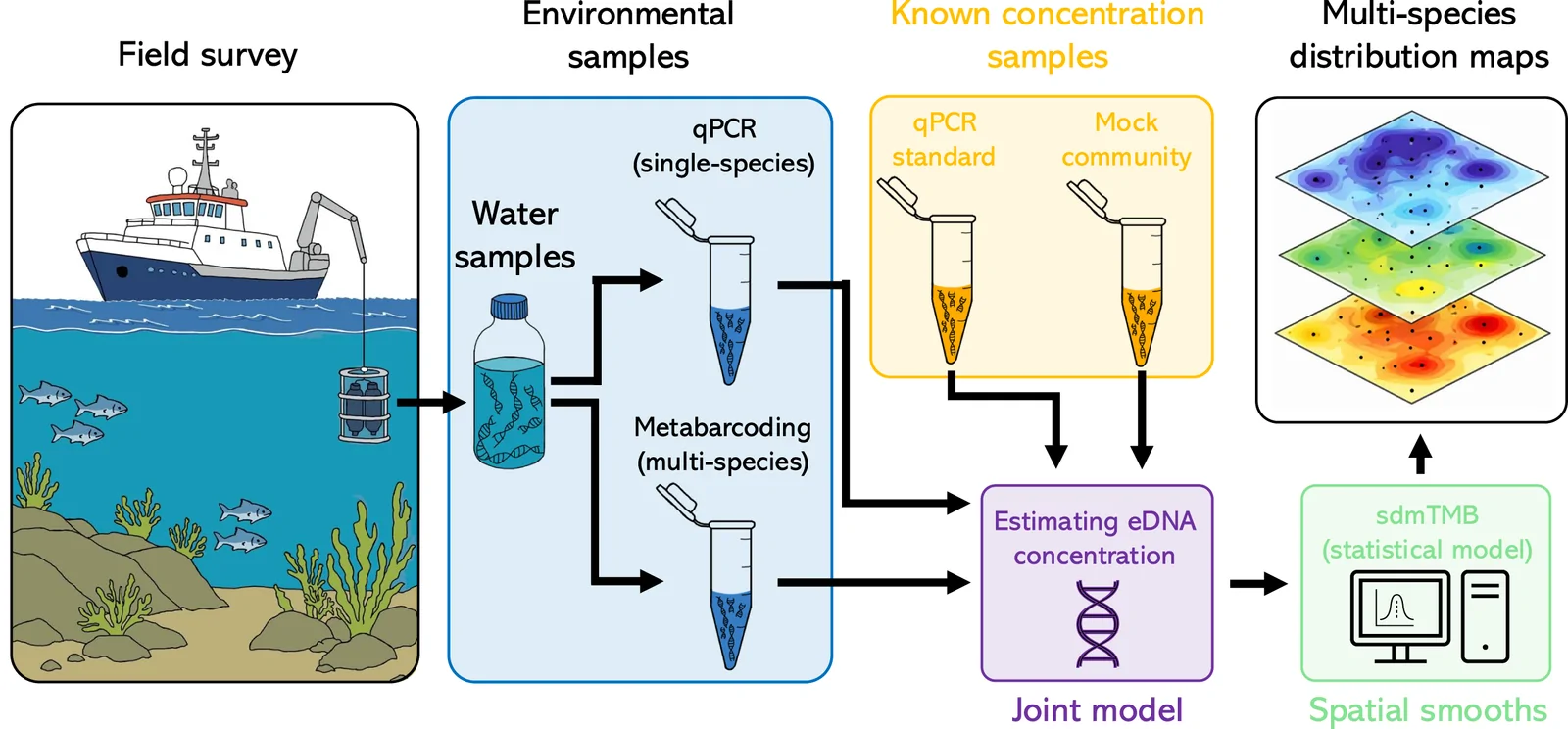
Conceptual workflow for estimating quantitative, multispecies eDNA abundances and mapping their three-dimensional distributions. Seawater collected at multiple depths during a field survey from which two molecular analyses (blue): single-species qPCR of Pacific hake, giving absolute DNA concentration, and multi-species MiFish-U metabarcoding, giving relative species abundance are conducted. Known-concentration samples (yellow) calibrate each stream—qPCR standards anchor the standard curve, and mock communities correct metabarcoding amplification bias. A joint Bayesian model (purple; for more see File S1 and Fig. S1) uses the hake concentration to convert metabarcoding proportions into absolute eDNA concentrations for all species, which are then spatially smoothed with sdmTMB (green) to produce three-dimensional distribution maps. All symbols and illustrations were drawn by the corresponding author.

## Methods

### Sample collection, processing, and quantitative PCR

Portions of this text were previously published as part of a preprint (https://doi.org/10.1101/2025.10.30.685180). We used seawater samples collected in 10 L Niskin bottles deployed on a CTD rosette from dusk to dawn at five depths (0, 50, 150, 300, 500 m) from stations along the West Coast of the US aboard the *Bell M. Shimada* during Summer 2019 (July–August) on the Joint U.S.-Canada Integrated Ecosystem and Pacific Hake Acoustic-Trawl Survey (de Blois, 2020). A total of 2.5 L of water samples were collected from each Niskin (*n* = 1,794) and immediately filtered on 47 mm diameter mixed cellulose ester (MCE) sterile filters with a 1 μm pore size and preserved in Longmire’s buffer until extraction. The extractions of DNA were done 1 month after the collection of samples using a phase lock phenol chloroform protocol and negative extraction controls (Longmire’s buffer) were included daily (File S1). The quantitative polymerase chain reaction (qPCR) was analyzed in triplicate using a qPCR assay (File S1), multiplexed for Pacific hake, sea lamprey, and eulachon, as described in Ramón-Laca, Wells & Park (2021) and Shelton et al. (2022). In this study a subset of those samples (*n* = 554; representing 543 unique station–depth combinations) was used for metabarcoding analysis in addition to qPCR samples already published for hake quantification in Shelton et al. (2022).

### Metabarcoding: environmental samples and mock community

All environmental samples were randomized and sequenced in seven Illumina MiSeq (v3 600 cycle kit) libraries. Total PCR reactions, including PCR blanks (*n* = 7) and positive controls (*n* = 7), were amplified using universal MiFish-U primers (Miya et al., 2015) (see File S1 for details on metabarcoding methods). Importantly, we meta-barcoded only one water sample (biological replicate) collected from each sampling station.

Sequences were de-multiplexed and the adapters were removed by Illumina processing software tools (Illumina, San Diego, CA, USA). Primers were removed with Cutadapt v4.9 (Martin, 2011). We then used DADA2 (default parameters; Callahan et al., 2016) to denoise sequences, remove chimeras, and generate amplicon sequence variants (ASV). The ASVs were then annotated against the NCBI nucleotide (nt) database (accessed January 2025) using BLASTn, applying cascading percent-identity thresholds (99.3%, 98%, then 96%). Each ASV was assigned to the least common ancestor of the top hits at the highest threshold that returned a match, yielding classifications at species, genus, family, or higher ranks. For all species targeted here, assignments resolved to the species level. Samples with fewer than 1,000 total reads were filtered as an indicator of poor reliability. Furthermore, samples lacking positive detection of Pacific hake (reference species) in both qPCR and metabarcoding datasets were removed from downstream analysis, due to the model dependence of the reference species being present in both (see File S1). Of the 554 initial environmental samples, 535 passed quality filtering and 371 (representing 358 spatially explicit locations in three dimensions, latitude, longitude, and depth) of those contained Pacific hake in both data streams (see Fig. S4). Details on the bioinformatic pipeline are available in File S1.

To calibrate metabarcoding observations and account for species-specific amplification bias (Shelton et al., 2023; Gold et al., 2023), we constructed multiple mock communities comprised of genomic DNA from a total of 39 species of fish. The validated DNA extracts or tissues were obtained from the University of Washington Fish Collection at the Burke Museum or the NOAA Northwest Fisheries Science Center collections. After quantifying the concentration of mitochondrial DNA template in each DNA extract, we constructed a total of eight mock communities of varying species compositions (see Table S1). We selected 12 species (additional to Pacific hake) of commercial and ecological importance that co-occurred in the environmental samples and in the mock community for downstream analysis: Pacific herring (*Clupea pallasii*), northern anchovy (*Engraulis mordax*), northern smoothtongue (*Leuroglossus stilbius*), Dover sole (*Microstomus pacificus*), Pacific sardine (*Sardinops sagax*), Pacific chub mackerel (*Scomber japonicus*), widow rockfish (*Sebastes entomelas*), northern lampfish (*Stenobrachius leucopsarus*), longfin dragonfish (*Tactostoma macropus*), blue lanternfish (*Tarletonbeania crenularis*), eulachon (*Thaleichthys pacificus*), Pacific jack mackerel (*Trachurus symmetricus*). After amplification and sequencing (File S1), we used mock communities (which were run under the same PCR conditions as environmental samples) to estimate species-specific amplification efficiencies and correct for amplification bias following.

### Statistical analysis

To generate estimates of DNA concentration for each of the 12 focal species, we constructed a joint statistical model for qPCR and Mifish-U metabarcoding observations from the same samples (Fig. 1). We first used qPCR observations of hake DNA concentration for all available water samples (1,794 unique water samples were assayed and a total of 5,394 qPCR reactions). This model component takes observations of the PCR cycle at which amplification was detected in each field sample and estimates a hake DNA concentration for each water bottle collected at each sampling station depth combination (Fig. S1). The statistical model accounts for sample-specific effects (*e.g*., dilution of the sample before PCR amplification) and the effect of replicate water samples (a random effect), to provide estimates of hake DNA concentration in from each water sample collected at each location and depth (Fig. S1). This qPCR component provides estimates of DNA concentration for Pacific hake that can then be linked to the multispecies proportions provided by DNA metabarcoding (File S1; Fig. S1).

Metabarcoding observations are the second component of the joint statistical model. Unlike qPCR, metabarcoding data are compositions, so each sample provides only information about species eDNA proportions rather than absolute DNA concentrations. For this model component, we followed Shelton et al. (2023) and constructed a compositional model for the metabarcoding data of 371 of the water samples analyzed (after quality filtering) for Pacific hake qPCR. We used the metabarcoding observations of the eight mock communities to calibrate the amplification efficiency of the 12 focal species (plus hake, the 13^*th*^ species). In the absence of qPCR data, this model would produce estimates of proportional DNA contributions for each water sample. For each sample in which we had nonzero estimates of hake DNA concentration from qPCR and nonzero estimate of hake proportional contribution from metabarcoding (Figs. S4 and S10), we then estimated the DNA concentration for each of the 12 remaining focal species (File S1). Therefore, our model generated estimates of the DNA concentration (copies per L) for each species. Hake DNA was detected by qPCR in 86.7% of the samples (883 out of 901 station-depth) and 67.7% of the metabarcoding observations. Samples lacking hake detections were sporadically distributed horizontally across the study area (Fig. S4), while vertically slightly more systematically. Surface (0 m) samples yielded the lowest proportion of hake-positive detections for both qPCR and metabarcoding (Fig. S10).

While alternative approaches exist for converting relative metabarcoding proportions to absolute abundances—such as internal spike-in standards (Ushio et al., 2018) or Unique Molecular Identifiers (UMIs; Hoshino et al., 2021)—we instead leverage existing qPCR-derived hake abundance estimates from Shelton et al. (2022). Additionally, we focused on species that were relatively common in the raw dataset, those well above the lower detection limits under our sampling and sequencing conditions, ensuring greater confidence in their consistent detectability. Although many more species were detected in the raw data, the majority were much rarer and thus less reliably observed.

The statistical model (Fig. S1) provides predictions of species-specific DNA concentrations at each location and depth sampled ( d = 0, 50, 150, 300, or 500 m). We used the sdmTMB package (Anderson et al., 2022) to generate smooth maps of DNA concentration for each species independently (Fig. S1, green box). As Pacific hake (*Merluccius productus*) has been examined elsewhere using qPCR (Shelton et al., 2022), we did not include the spatial patterns of that species in our analysis.

Direct comparison of our eDNA-derived patterns against independent estimates or observations of fish biomass falls beyond the scope of this study, particularly given the lack of observational or repository data for many species included here. Consequently, we cannot quantify the method’s uncertainty relative to biological signals observed by other gear types (*i.e*., fish counts or acoustic detections) since such data are currently unavailable and are themselves subject to different biases. Instead, we report both the standard error of the spatially smoothed predictions and the spread of posterior distributions of the eDNA estimates from the joint model as relative measures of uncertainty.

Analyses, figure preparation, and data curation were performed in R (R Core Team, 2024) and the joint statistical model for qPCR and metabarcoding was coded in stan (Stan Development Team, 2023). The convergence of the statistical model (MCMC) was assessed for all model parameters and are reported in model convergence in File S1. All data and code can be found in https://zenodo.org/records/20754663 and https://github.com/gledguri/Quantitative-Multispecies-eDNA.

## Results

We estimated the spatial eDNA distributions of 12 ecologically important fish species across ten degrees of latitude between the surface and 500 m deep (Figs. 2, S2). These maps included commercially important and well-known coastal-pelagic species (northern anchovy, Pacific sardine, Pacific herring, jack mackerel) as well as other high-biomass species otherwise lacking well-described distributions (*e.g*., widow rockfish, northern lampfish; Fig. 2). Our analyses presented detailed, depth-specific information on eDNA abundance—likely a proxy for the abundance of the fish themselves—that is difficult to obtain with traditional sampling methods. In the following, we highlighted results for six species (Fig. 2) and compared results from eDNA with available distribution and life-history information from the scientific literature. Distribution maps for all remaining species are presented in Fig. S2. We then compared distributions among species (Fig. 3) and examined emergent multispecies patterns and richness derived from eDNA (Fig. 4).

**Figure 2:**
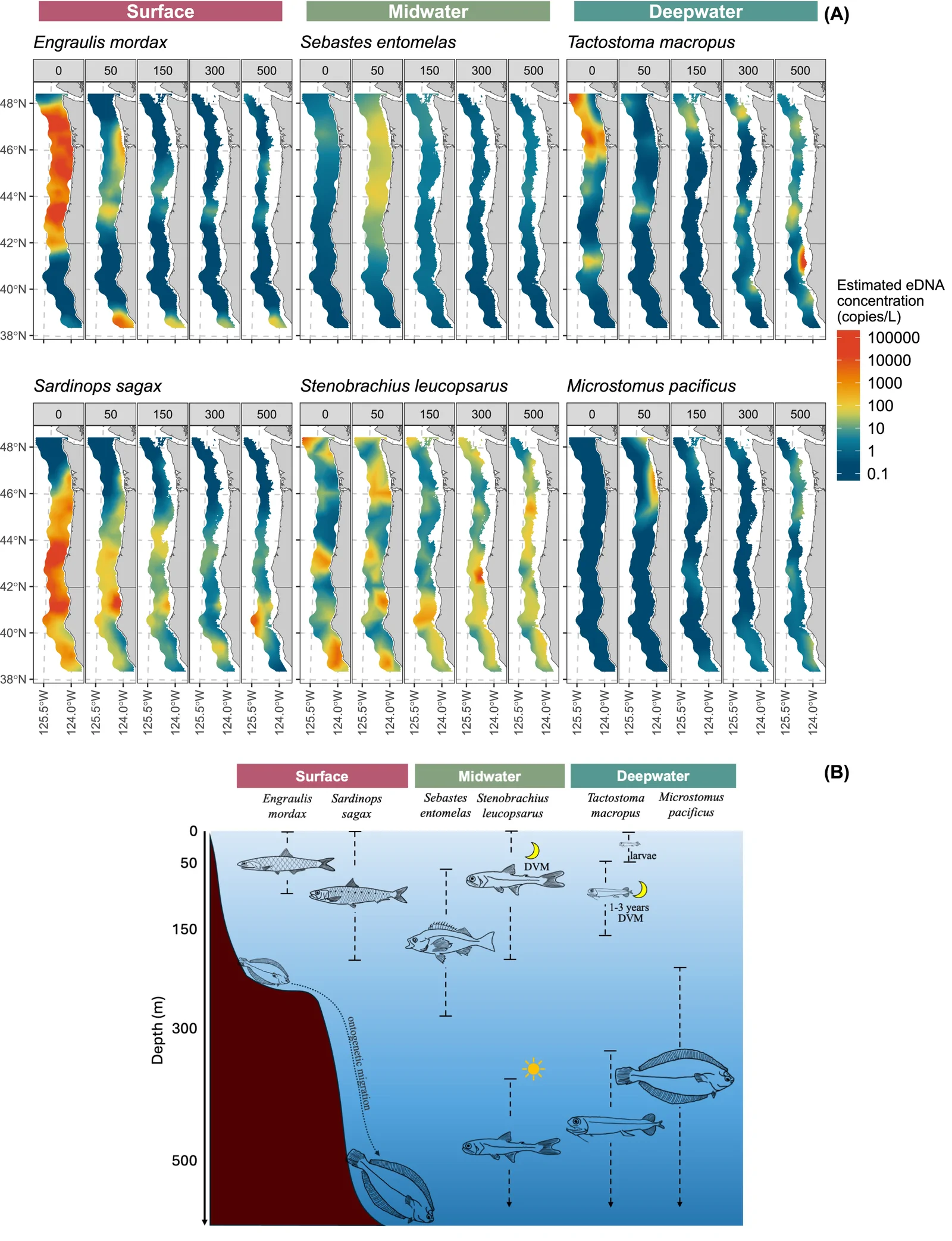
US West Coast estimated eDNA concentrations and literature depth distributions. Presented here are only six species (for the remaining six other species, see Fig. S1). The concentrations are estimated for each depth 0, 50, 150, 300, and 500 m shown in (A) and the depth distribution of these species from the literature shown in (B).

**Figure 3:**
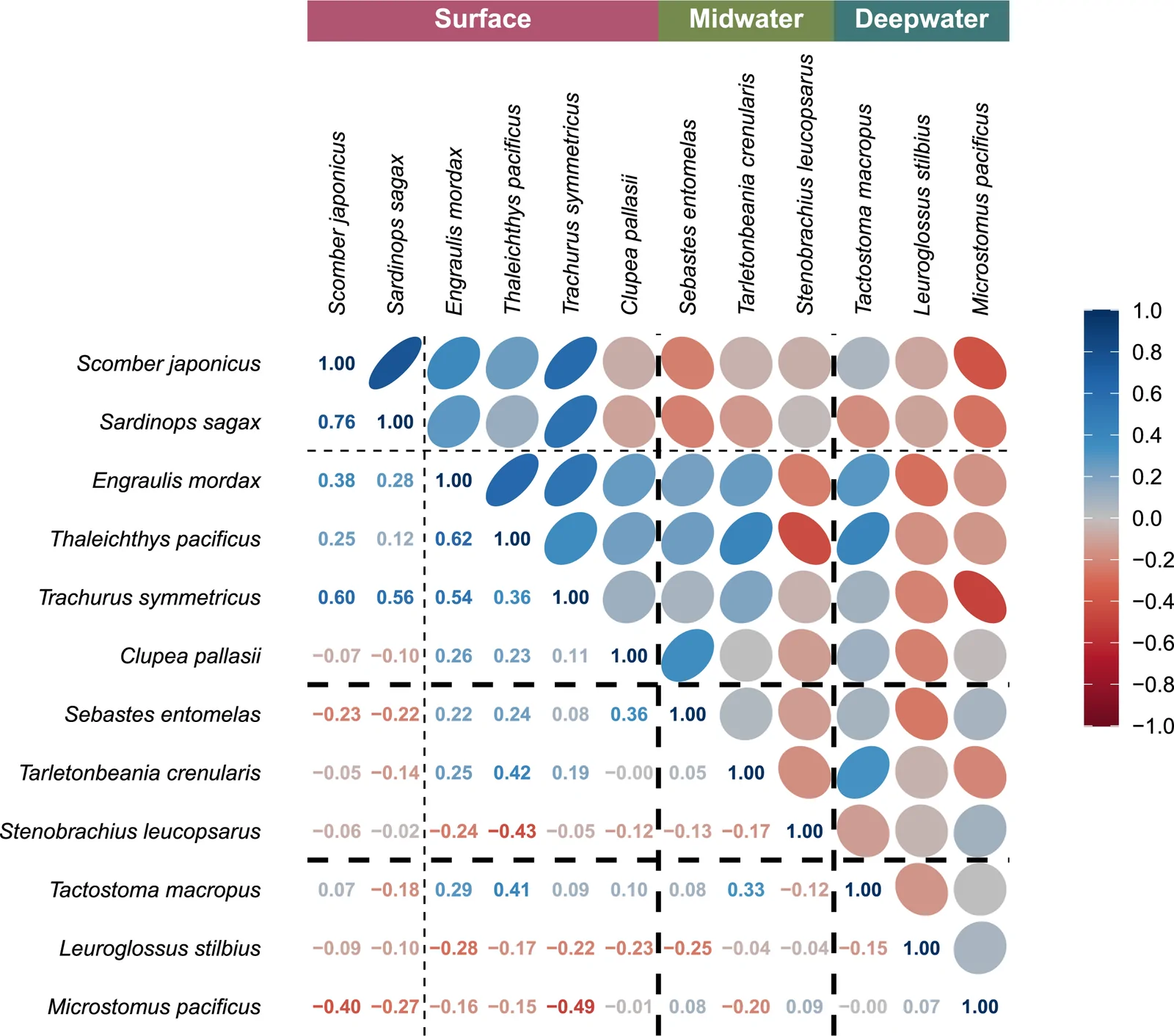
Correlation plot describing species co-abundance and co-occurrence. Spearman’s correlation coefficient is expressed in numbers and colors ranging from −1 (red) negatively correlated to 1 (blue) positively correlated. Species are ordered in ecological groups (surface, midwater, and deepwater) to visually indicate the gradual change of species co-occurrence.

**Figure 4:**
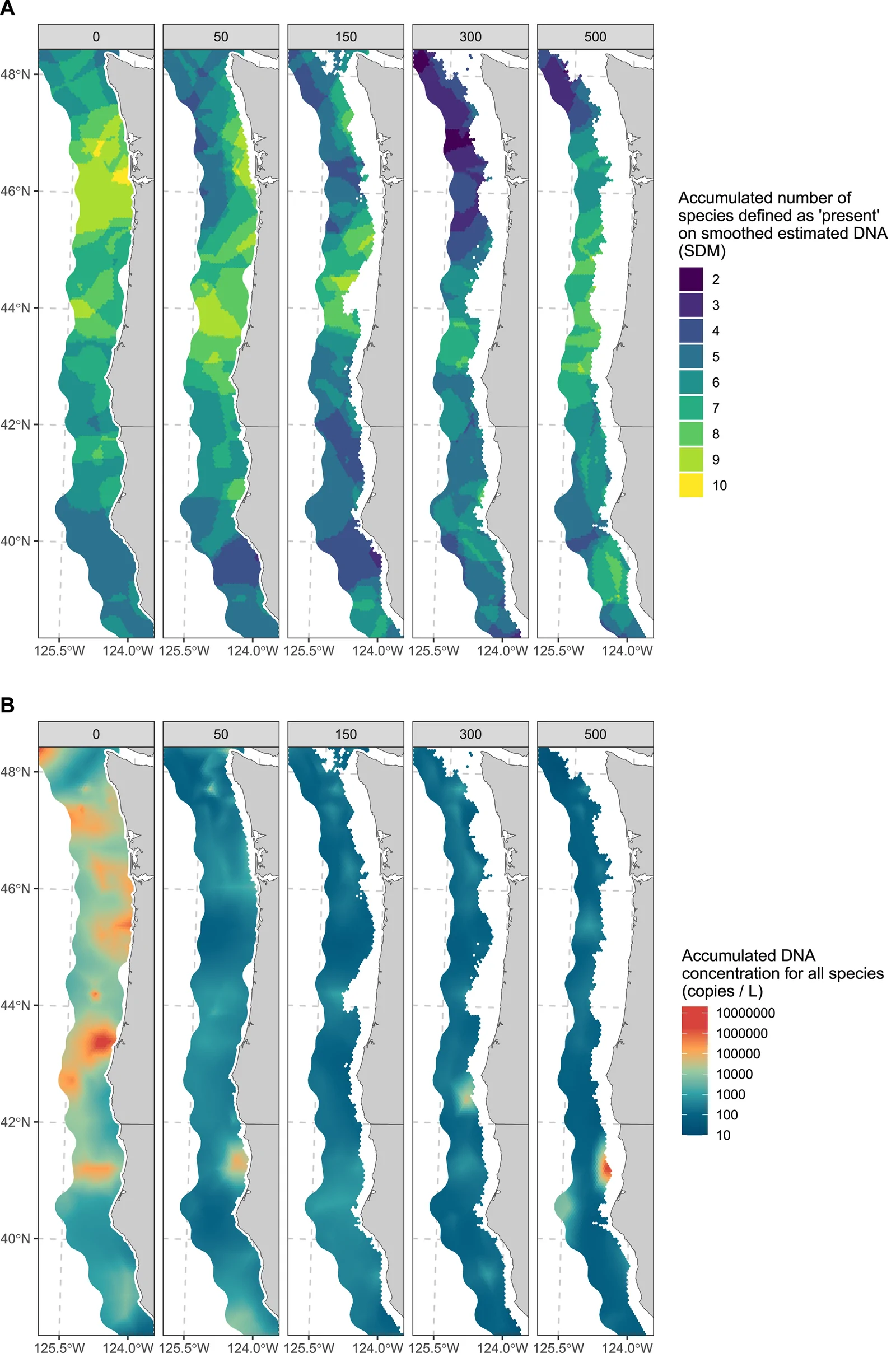
Spatial and vertical distribution of species richness and cumulative environmental DNA concentration. Species richness (A) represented as the cumulative number of species present (species with more than 1.5 copies/L (see Fig. S2 for defining presence) in smoothed estimated eDNA concentrations) and accumulated eDNA concentration (B) represented as the cumulative sum of eDNA (copies/L) across all species.

### Surface species

Northern anchovy (*Engraulis mordax*) and Pacific sardine (*Sardinops sagax*) are two iconic coastal pelagic species of the eastern North Pacific (Steinbeck, 1945) and play a vital ecological role linking lower and upper trophic levels (*e.g*., diets for Chinook salmon and sea lions; Kaplan et al., 2019); historically, these supported large commercial fisheries, particularly in California. Although larvae and juveniles typically occupy the upper 50 m, adults move to 100 m during the day to avoid predators (Litz et al., 2008). Our eDNA estimates consistently reflected the expected three-dimensional distributions of known surface-dwelling species, with high concentrations in surface waters for both species (Litz et al., 2008; Parnel, Emmett & Brodeur, 2008) and particularly near the Columbia River outflow (46.2°N; Fig. 2). The literature suggests that sardine favors warmer and more southerly waters than anchovy (SST range: 13–24 °C for sardine *vs*. 10–14 °C for anchovy; Litz et al., 2008; Sato et al., 2018). The eDNA patterns tracked these differences with sardine eDNA in greatest abundance at 41°N (range 40°–45°), while anchovy eDNA abundance was greatest at 43.5°N (range 42°–48°; Fig. 5) suggesting that eDNA captured subtle but meaningful differences in habitat preferences. Lastly, sardine eDNA showed occasional high concentrations below depths of 150 m—contrary to previous reports that indicated only occasional detections at these depths (Zwolinski & Demer, 2024). However, these detections were rare, with only 2 of 120 samples with estimated concentrations greater than 100 copies/L, suggesting that the deep signal likely represents unusual observations perhaps resulting from deposition *via* feces, passive settling, carcass decay, or transient individuals.

**Figure 5:**
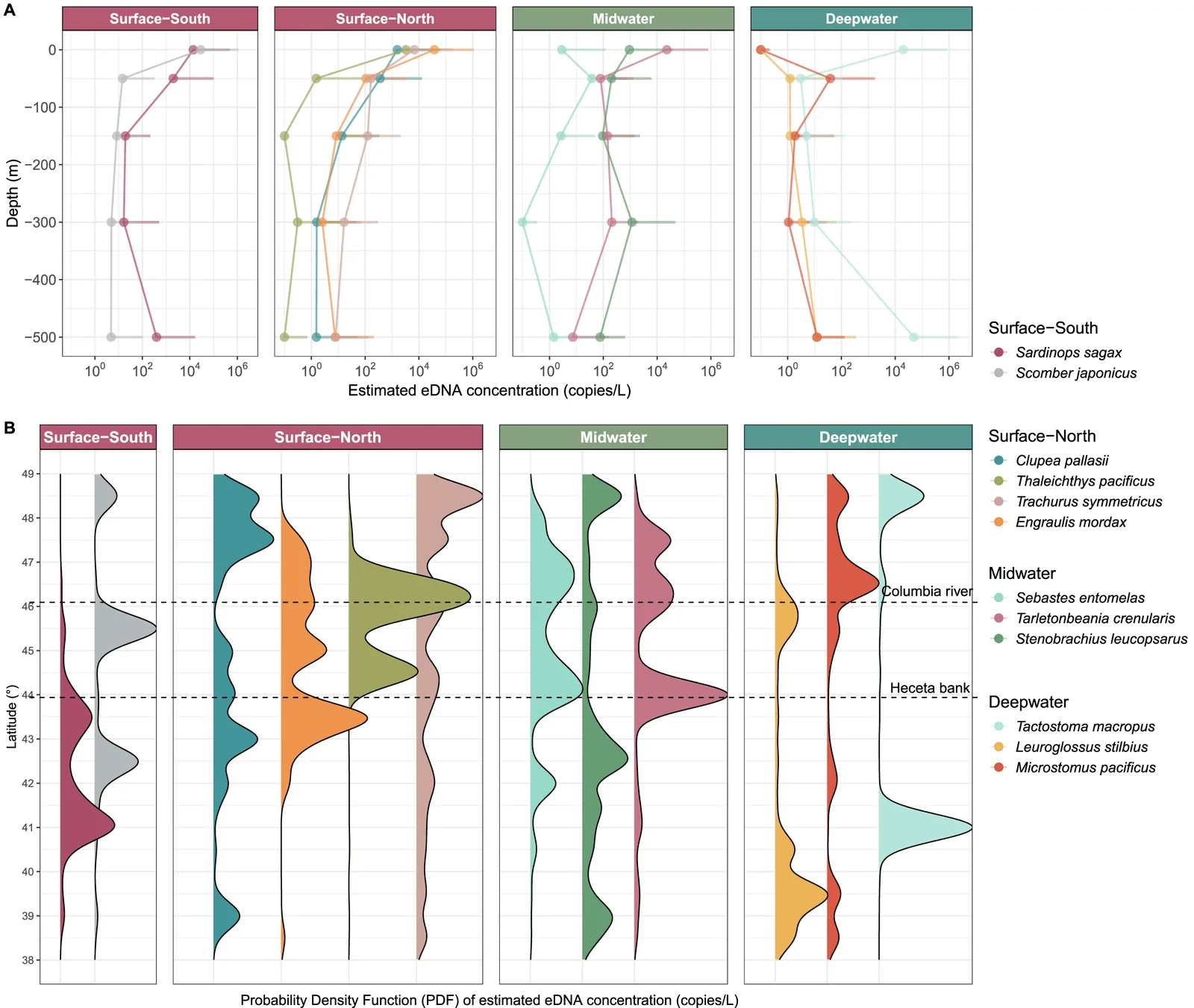
Vertical and latitudinal distribution of eDNA concentrations grouped into ecological groups (surface south, surface north, mid-water and deep-water). The mean eDNA concentration in vertical distribution is shown with dots and the 99% upper quantile with ticks (A). Probability density functions (PDF) of estimated eDNA concentrations across latitudinal gradients (B; square root transformed of the x-axis to enhance low-concentration peaks). (B) Probability density functions; hence the x-axis represents probability density (unitless), scaled such that the area under each curve integrates to 1. Key geographic features, such as the Columbia River and the Heceta Bank, are marked to indicate the alignment of species distributions.

### Midwater species

Widow rockfish (*Sebastes entomelas*) is common between 100–350 m from northern Baja California to southern Alaska, with pelagic larvae and juveniles generally detected in shallower habitats (<150 m depth; Reynolds, 2001; Bosley et al., 2014) including nearshore kelp forests (Laidig, Chess & Howard, 2007; Quigley et al., 2024). Furthermore, because it is a common bycatch species in the Pacific hake fishery, concerns about bycatch of widow rockfish can also influence the much larger hake fishery (*i.e*., it is a ‘choke’ species for the hake fishery; Somers et al., 2018). We find widow rockfish eDNA strongly and nearly exclusively associated with sample depths of 50 m (Figs. 2 and 5), consistent with pelagic larvae and juveniles reported in the literature, though somewhat shallower than the depths reported for adults (Reynolds, 2001; Bosley et al., 2014; Quigley et al., 2024). Given that the water samples were taken at night, this pattern potentially reflects the behavior of the nocturnal feeding schooling of the species (Reynolds, 2001; Bosley et al., 2014).

The eDNA patterns for northern lampfish (*Stenobrachius leucopsarus*)—an abundant myctophid that migrates from 400–700 m up to 20–200 m at night (Moku et al., 2000; Suntsov & Brodeur, 2008)—are again consistent with the ecology of the species, having notably lower concentrations in surface samples. Most likely due to the diel vertical migration of the northern lampfish, the signal of eDNA for this species is observed across depths.

### Deep-water species

Among deeper water fish, eDNA revealed spatial and potentially ontogenetic migration patterns for species that are common, yet understudied, such as the longfin dragonfish (*Tactostoma macropus*). The available literature suggests that dragonfish occur roughly between 40–50°N (adults at 300–900 m, larvae at 0–60 m in summer; Kawaguchi & Moser, 1993) and perform vertical diel migrations to around 100 m (Willis & Pearcy, 1982; Kawaguchi & Moser, 1993). eDNA concentrations appeared to reflect this activity (Fig. 5), with the predominant surface signal at northern latitudes likely reflecting larvae and juveniles (Fig. 2; near Columbia River discharge), and two deeper hotspots at 300 and 500 m separated by about eight degrees of latitude. However, there are no other quantitative estimates of the abundance or detailed distribution patterns of dragonfish in the literature.

The estimates of eDNA concentrations for Dover sole are also consistent with known habitats. This benthic species lives at 200 to 1,200 m along the Pacific coast (Brodziak & Mikus, 2000; Drazen, 2007; Drazen & Haedrich, 2012) with individuals moving progressively deeper as they grow (Hunter et al., 1990; Vetter et al., 1994). High eDNA concentrations were found almost entirely in water samples collected adjacent to the seabed (Fig. 2; Ono et al., 2016).

### Species associations

Species with similar ecological traits and habitat preferences had correlated eDNA concentrations (Fig. 3). Pairwise correlations of eDNA concentration showed meaningful patterns among species associated with depth (Fig. 3). Surface-associated species were nearly all positively correlated with one other, with some particularly strong correlations (Pearson’s ρ>0.50; Fig. 3). The correlations were weak among the six mid- and deepwater species (all $\rho < \left| {0.40} \right|$; Fig. 3). Within surface species, chub mackerel and sardine showed very high correlation ( ρ=0.76; Fig. 3) with both more common in the more southern waters (Figs. 2 and 5). The concentrations of the remaining surface-dwelling species (northern anchovy, eulachon, jack mackerel, and Pacific herring) had modest correlations with midwater species (Fig. 3) consistent with a more northerly distribution (Fig. 5). Together, these findings suggested that eDNA captures both depth and latitudinal habitat associations. The patterns of deepwater species were distinct (Figs. 2 and 5) and are not strongly correlated with those of other zones (Fig. 3). Deeper water tends to feature lower and more diffuse fish eDNA concentrations (Figs. 2 and 5) hence deep-water samples may make similarities among species distributions more difficult to detect at 300 m and deeper.

### Species richness and total eDNA accumulation

The eDNA abundance of individual species can be combined to interrogate important aggregate ecological patterns—again, with the caveat that spatial patterns of eDNA do not necessarily reflect patterns of adult biomass. For the 12 species considered here, we showed how species richness (see Figs. S3 and S9 to define species presence) from eDNA varied substantially and predictably with depth, with the highest richness observed at the surface (Fig. 4A). In part, this pattern is likely to be an artifact of the focal species included in this study, chosen for their relevance to fisheries management and for their frequency in the observations (see Methods), but the richness-depth gradient also reflected the expectation that habitat and nutrient availability is greatest near the surface (Smith & Brown, 2002; Hickey et al., 2005). Surface waters also host the eggs, larvae, and juvenile stages of many meso- and bathypelagic species (Parnel, Emmett & Brodeur, 2008), and hence we both expected and observed higher richness in the upper layer of the water column (Kim & Barth, 2011).

Species richness also strongly corresponded to the influence of the Columbia River plume (Fig. 4A), which extends well beyond the coastline (Hickey & Banas, 2003). Nitrate-rich river water interacts with ocean currents and intensifies coastal upwelling, enhancing both primary and secondary productivity (Hickey et al., 2010) and likely influencing the patterns of species richness we observe (Fig. 4A; Tolimieri et al., 2015). The total accumulation of fish eDNA (Fig. 4B) suggested the highest aggregation of forage fish DNA near Heceta Bank (44 degrees N; overlapping anchovy and sardine distributions are the two largest contributors to the observed peak of accumulated eDNA concentration). In addition to surface dwelling species, the accumulation of fish eDNA revealed a high aggregation of two important mesopelagic fishes (*Sebastes entomelas* and *Tarletonbeania crenularis*)—a center of abundance for commercial fisheries (Tissot et al., 2008; Bosley et al., 2014). Although our eDNA-derived richness reflected an index based on a subset of taxa (12 species here) and integrates signals over space and time, its spatial patterns closely corresponded to known ecological structures and independently observed biodiversity hotspots.

### Uncertainty of eDNA estimates

Consistent with our expectation, both smoothed standard error and standard deviations of concentration estimate posterior distributions were the lowest in regions with the high estimated eDNA concentrations and *vice versa* (File S2). These patterns indicated that areas with positive detections and high concentrations of eDNA yielded more precise and reliable predictions for species distributions (Fig. S9), while negative detections were associated with substantially greater uncertainty because the model could not constrain the exact magnitude of low concentration (Fig. S9).

## Discussion

We report 12 species-specific DNA concentrations of fish over ten degrees of latitude and across the continental shelf to 500 m, suggesting generalizable pathways for the broader use of eDNA in both fisheries and ecosystem management.

### eDNA as a management tool

The spatial patterns derived from eDNA generally appear to reflect the known distributions and ecological associations of a diverse suite of fish species along the California current. Consistent with previous studies that have demonstrated species-specific relationships between eDNA concentrations and fish biomass—such as those of Salter et al. (2019) for Atlantic cod, Shelton et al. (2022) for hake, Maes et al. (2023) for two flatfishes, and Guri et al. (2024) for multiple fishes—we find species-level eDNA concentrations align closely with the physiological constraints of preferred habitats (*e.g*., temperature tolerance or food availability) described for each species in the available literature. The observed latitudinal differences in eDNA concentration between species—such as those between anchovy and sardine—highlight the thermal ranges of the species and are largely consistent with other surveys (Zwolinski & Demer, 2024); furthermore, eDNA patterns appear to reflect vertical distributions of species consistent with the diel or ontogenetic movement of the species between surface and deep water habitats. Additionally, emergent patterns of species richness and estimated eDNA aggregation correspond with known ecological drivers, evidence that eDNA traces can reliably capture ecologically meaningful patterns (Fig. 4) as we identified aggregations of richness and total eDNA near high-productivity regions of ecological and commercial value. By mapping three-dimensional spatial patterns, eDNA provides information on species distributions, habitat preferences, and ecological processes that is often otherwise unavailable. However, a few deviations from expected patterns are also apparent, most notably the presence of sardine eDNA at depths exceeding 150 m, where individuals are rarely observed. Such signals likely reflect downward transport processes (*e.g*., particle sinking, fecal deposition, or carcass decay).

For well-studied taxa, eDNA can augment existing information to help refine insights on habitat preferences, seasonal patterns, or temperature tolerances. For poorly studied species, eDNA serves as an exploratory tool, detecting elusive taxa and revealing unexpected habitats. Widow rockfish, for example, appear almost exclusively in pelagic habitats around 50 m deep, which are sampled poorly by traditional bottom-trawl surveys (Keller, Wallace & Methot, 2017). This species is of special interest, as a targeted commercial harvest resumed in the late 2010s after a decade of closure (harvest was less than 500 mt annually between 2003 and 2013 but has exceeded 10,000 mt annually since 2018), and no existing survey adequately captures its patterns of abundance (Adams et al., 2019).

### Advantages and limitations

Determining the abundance and distribution of marine species is challenging, as different taxa occupy distinct habitats and respond differently to each type of sampling gear, producing variable detectability and catchability in traditional multispecies surveys (Zhang et al., 2020). Metabarcoding can circumvent these limitations by effectively sampling numerous taxa simultaneously with a single noninvasive ‘gear’ type, offering a standardized, scalable and replicable approach for broad spatial and temporal surveys; however, it remains unclear how the concentrations of eDNA are related to the biomass of the fish themselves at relevant spatial scales. To the extent that eDNA is reflecting species habitat preferences and known distributions at the scale of tens to hundreds of kilometers, our estimates can have direct relevance for the management of natural resources on large scales. For example, unitless indices of abundance play a central role in determining stock status for fisheries applications (*i.e*., stock assessments in the US under the Magnuson-Stevens Act) as well as informing conservation status (*e.g*., under the US Endangered Species Act). Recent regulatory analyses indicate that eDNA-derived data can meet the information requirements of these statutes (Laschever et al., 2023), suggesting that abundance indices from eDNA could help satisfy a variety of existing management requirements (Baetscher et al., 2025). Although these indices are still in their infancy and will require additional technical developments to ensure their proper usage (accounting for transport, degradation, and shedding rates), they share key conceptual foundations with abundance estimates derived from more traditional ocean survey sampling methods (*i.e*., net-based or acoustic surveys; Salter et al., 2019; Shelton et al., 2022; Maes et al., 2023; Guri et al., 2024).

The observable eDNA signal at any given location and time is an integrated product of DNA production and loss across time and space (Andruszkiewicz et al., 2019; Gold et al., 2023). In some contexts, it may be appropriate to aggregate spatially proximate samples to better reflect the combined forces of eDNA production, transport, and decay (Guri et al., 2024). As in any sampling context, individual samples may over- or underrepresent the parameter of interest due to sampling variability, transport, and other mechanisms. These stochastic effects are likely to average out at larger scales and are directly akin to the observation variability present in traditional sampling methods (*e.g*., net or active acoustics).

Furthermore, since DNA production from the source is continuous and relatively localized in both spatial dimensions (within km; Baetscher, Nuetzel & Garza, 2023) and temporal (within hours; Strickler, Fremier & Goldberg, 2015; Mächler, Osathanunkul & Altermatt, 2018; Collins et al., 2018; Allan et al., 2021), the distribution of eDNA from a given species at any given time will be smoother and more extensive than the distribution of discrete individuals. Such characteristics establish eDNA as a source of continuous and integrated biological information that extends beyond the precise sampling location and timeframe. Due to the integrated nature of the eDNA signal and (here) the subsequent model-based smoothing, our findings are best interpreted as representing large-scale ecological patterns rather than localized dynamics.

Vertical eDNA signals in this study largely align with the known depth distributions of most species and represent a dimension that most traditional survey methods do not easily capture (*i.e*., co-located samples at multiple depths). For instance, shallow-water species (sardine, anchovy, herring) occur largely at the surface and 50 m depth bins; widow rockfish occurs almost exclusively at 50 m; species found throughout the water column are either diel vertical migrators or species occupying different depths at various life history stages (Fig. 4B); and bottom-dwelling fishes are detected primarily at the deepest point sampled on any station. However, interpreting these patterns is complicated by multiple factors. First, vertical transport processes, especially downward eDNA flux *via* particle settling, can obscure biological signals at depth. However, if downward transport were a pervasive mechanism, we would expect to observe (i) a monotonic increase in eDNA species richness with depth, and (ii) a lack of depth-specific species patterns in the dataset. Instead, we observe the opposite trend in richness (it decreases with depth), and a variety of depth-specific patterns—most notably widow rockfish (*Sebastes entomelas*), which is closely associated with the 50 m depth bin. We note that species with lower overall concentrations, such as the widow rockfish, may also appear to have a restricted eDNA distribution because any downward-transported signal would be diluted below the limit of detection.

Second, fecal deposition from predators, combined with sinking carcasses and passive particle settling, could all contribute to the observed deep eDNA signal—such as the sporadic deep sardine detections. These mechanisms are not mutually exclusive and may operate simultaneously, and we cannot empirically distinguish among these mechanisms with the current data.

For other pelagic species that undergo ontogenetic or diel vertical migrations (DVMs), disentangling biological signals becomes more challenging. At present, eDNA cannot identify life stages or pinpoint shedding times and therefore differentiating DNA derived from organismal migration or the passive transport of residual DNA is not currently possible. Despite these limitations, marine communities can be clustered by depth through eDNA surveys (Jeunen et al., 2019; Easson et al., 2020), and eDNA can reach quasi-equilibrium within days under stable migration patterns (Andruszkiewicz Allan et al., 2021). This stability supports linking eDNA depth distributions to resident depths (day/night) if migration consistency is assumed. In summary, the overall observed depth-specific pattern of eDNA generally aligns with literature reports and known species depth preferences; however, detailed interpretations require caution and should consider the confounding effects of vertical eDNA transport.

A further caveat concerns the amplification-bias correction itself. Amplification bias is a well-documented limitation of quantitative metabarcoding. Using mock communities of known composition, Shaffer et al. (2025) found that species-specific amplification efficiencies vary widely among species, primer sets, and polymerases. Empirical calibration is therefore required, and we accordingly estimated species-specific efficiencies against mock communities before translating reads into quantitative abundances. The resulting eDNA distributions were broadly consistent with known species niches and depth preferences, which suggests that the correction performed as intended but does not isolate its contribution from that of the underlying uncorrected metabarcoding data. Carrying corrected and uncorrected estimates through identical ecological endpoints would provide a direct test and clarify the magnitude that the calibration meaningfully alters survey-scale inference. However, Shaffer et al. (2026), using the same primer set and calibration framework applied here, found that bias correction substantially changed the inferred community composition, and that the corrected concentrations agreed closely with independent droplet digital PCR measurements.

A notable limitation of our approach is its dependence on Pacific hake as the single reference species. Because all species-level eDNA concentration estimates are derived by scaling metabarcoding proportions against hake-derived absolute concentrations, any error or bias in the hake qPCR estimate propagates across all other species in the community-level output (Fig. S9B). Additionally, for samples where the reference species was not detected (either by qPCR or metabarcoding) the joint model cannot estimate the eDNA concentrations for the remaining (multi) species, posing a limitation on the spatial inference. Moreover, sampling effort was balanced across depth bins overall (given the bathymetry, Fig. S10). However, detections within the surface layer were less even. Surface samples (0 m) in the southern latitudes (38°–40°N) had fewer detections than elsewhere, so smoothed distribution maps for that region rest on fewer observations and should be interpreted with caution (Figs. S4 and S11). Future implementations of this framework would benefit from incorporating multiple reference species with independent quantitative assays, which could reduce the leverage effect of a single species and improve the sampling distribution coverage where the reference species is less consistently detected.

## Conclusions

Multi-species eDNA, coupled with quantitative calibration, can produce management-relevant, three-dimensional patterns of fish distributions at large spatial scales. Molecular patterns alone are unlikely to drive quantitative natural-resources decisions, in part because eDNA generally does not provide auxiliary information important for managing populations (*e.g*., information on size- or age structure, fecundity, sex, or disease) and because translating eDNA concentration into biomass requires external data and species-specific proportionalities that may differ among taxa. We therefore view eDNA not as a replacement for established survey programs, but as a complementary data stream that can strengthen and extend the traditional surveys, by adding depth distribution information, helping fill gaps for poorly sampled taxa, and enabling integrated inference when combined with traditional observations.

## Supplemental Information

10.7717/peerj.21810/supp-1 Supplemental Information 1 Extended laboratory and statistical methods.

10.7717/peerj.21810/supp-2 Supplemental Information 2 Directed acyclic graph (DAG) of the joint Bayesian model structure to produce species eDNA distributions. Hake eDNA qPCR observations (blue) are informed by known-concentration qPCR standards (orange) to estimate hake DNA concentration. Additionally, the initial proportions (pre-PCR) of 12 species from metabarcoding are estimated using a separate mock-community model (orange) that accounts for amplification efficiency. These are jointly estimated through the model (purple), from which eDNA concentrations for all 12 species are derived and thereafter fed to a separate smoothing model (green). Colors match those in Figure 1. The notation for parameters, subscripts, and observations is indicated in gray.

10.7717/peerj.21810/supp-3 Supplemental Information 3 US West coast estimated eDNA concentrations and literature depth distributions. Presented here are the remaining six species (not included in Figure 1 due to space). The concentrations are estimated for each depth 0, 50, 150, 300, and 500 m shown in panel A and the depth distribution of these species from the literature shown in panel B.

10.7717/peerj.21810/supp-4 Supplemental Information 4 Frequency distribution of eDNA concentration for each species included in this study (n = 12). The dashed red line indicates the threshold model estimated concentration (copies/L) for defining presence and absence of sequenced (through metabarcoding) taxa.

10.7717/peerj.21810/supp-5 Supplemental Information 5 Three-dimensional distribution of samples collected that were run through qPCR (light blue), metabarcoding (dark blue), and were used jointly in the Bayesian model (red). Jointly modeled samples are those that detected Pacific hake—used as a reference species for quantification – in both qPCR and metabarcoding assays.

10.7717/peerj.21810/supp-6 Supplemental Information 6 Initial mock community proportional abundances. The initial proportional abundances were measured by amplifying the 12S rRNA gene using MarVer1 primers (Valsecchi et al., 2020) and ddPCR QX200 Droplet Digital PCR system (Bio-Rad, Inc.)) of the selected species (*n* = 12) across different mock communities (*n* = 8). NA represent no DNA template included.

10.7717/peerj.21810/supp-7 Supplemental Information 7 Uncertainty of species eDNA concentration estimation through the model and smoothing residuals.

10.7717/peerj.21810/supp-8 Supplemental Information 8 MIQE Checklist.

## Additional Information and Declarations

### DNA Deposition

The following information was supplied regarding the deposition of DNA sequences:

The raw sequence data are available at NCBI BioProject: PRJNA1426049.

### Data Availability

The following information was supplied regarding data availability:

The data and code are available at GitHub and Zenodo:

- https://github.com/gledguri/Quantitative-Multispecies-eDNA

- Guri, G. (2026). Quantitative eDNA abundances for multispecies monitoring along the US West Coast. https://doi.org/10.5281/zenodo.20754663.

## References

[ref-1] Adams GD, Kapur M, McQuaw K, Thurner S, Hamel O, Stephens A, Wetzel C (2019). Stock assessment update: status of widow rockfish (Sebastes entomelas) along the U.S. West Coast in 2019.

[ref-2] Allan EA, DiBenedetto MH, Lavery AC, Govindarajan AF, Zhang WG (2021). Modeling characterization of the vertical and temporal variability of environmental DNA in the mesopelagic ocean. Scientific Reports.

[ref-3] Allan EA, Kelly RP, D’Agnese ER, Garber-Yonts MN, Shaffer MR, Gold ZJ, Shelton AO (2023). Quantifying impacts of an environmental intervention using environmental DNA. Ecological Applications.

[ref-4] Anderson SC, Ward EJ, English PA, Barnett LAK, Thorson JT (2022). sdmTMB: an R package for fast, flexible, and user-friendly generalized linear mixed effects models with spatial and spatiotemporal random fields.

[ref-5] Andres KJ, Lodge DM, Andrés J (2023). Environmental DNA reveals the genetic diversity and population structure of an invasive species in the Laurentian Great Lakes. Proceedings of the National Academy of Sciences of the United States of America.

[ref-6] Andruszkiewicz EA, Koseff JR, Fringer OB, Ouellette NT, Lowe AB, Edwards CA, Boehm AB (2019). Modeling environmental DNA transport in the Coastal Ocean using Lagrangian particle tracking. Frontiers in Marine Science.

[ref-7] Andruszkiewicz Allan E, Zhang WG, Lavery AC, Govindarajan AF (2021). Environmental DNA shedding and decay rates from diverse animal forms and thermal regimes. Environmental DNA.

[ref-8] Baetscher DS, Nuetzel HM, Garza JC (2023). Highly accurate species identification of Eastern Pacific rockfishes (Sebastes spp.) with high-throughput DNA sequencing. Conservation Genetics.

[ref-9] Baetscher DS, Omori KL, Goethel DR, Shelton AO, Berger AM, Ledger KJ, Nichols KM, Larson WA (2025). The pragmatic sceptic: a practical approach for integrating environmental DNA into marine stock assessment and fisheries management. Fish and Fisheries.

[ref-10] Baldigo BP, Sporn LA, George SD, Ball JA (2017). Efficacy of environmental DNA to detect and quantify brook trout populations in headwater streams of the Adirondack Mountains, New York. Transactions of the American Fisheries Society.

[ref-11] Bosley KL, Miller TW, Brodeur RD, Bosley KM, Van Gaest A, Elz A (2014). Feeding ecology of juvenile rockfishes off Oregon and Washington based on stomach content and stable isotope analyses. Marine Biology.

[ref-12] Brodziak J, Mikus R (2000). Variation in life history parameters of Dover sole, *Microstomus pacificus*, off the coasts of Washington, Oregon, and northern California. Fishery Bulletin.

[ref-13] Callahan BJ, McMurdie PJ, Rosen MJ, Han AW, Johnson AJA, Holmes SP (2016). DADA2: high-resolution sample inference from Illumina amplicon data. Nature Methods.

[ref-14] Collins RA, Wangensteen OS, O’Gorman EJ, Mariani S, Sims DW, Genner MJ (2018). Persistence of environmental DNA in marine systems. Communications Biology.

[ref-15] de Blois S (2020). The 2019 joint U.S.-Canada integrated ecosystem and pacific hake acoustic-trawl survey: cruise report SH-19-06.

[ref-16] DiBattista JD, Fowler AM, Riley IJ, Reader S, Hay A, Parkinson K, Hobbs J-PA (2022). The use of environmental DNA to monitor impacted coastal estuaries. Marine Pollution Bulletin.

[ref-17] Doi H, Uchii K, Takahara T, Matsuhashi S, Yamanaka H, Minamoto T (2015). Use of droplet digital PCR for estimation of fish abundance and biomass in environmental DNA surveys. PLOS ONE.

[ref-18] Drazen JC (2007). Depth related trends in proximate composition of demersal fishes in the eastern North Pacific. Deep Sea Research Part I: Oceanographic Research Papers.

[ref-19] Drazen JC, Haedrich RL (2012). A continuum of life histories in deep-sea demersal fishes. Deep Sea Research Part I: Oceanographic Research Papers.

[ref-20] Easson CG, Boswell KM, Tucker N, Warren JD, Lopez JV (2020). Combined eDNA and acoustic analysis reflects diel vertical migration of mixed consortia in the Gulf of Mexico. Frontiers in Marine Science.

[ref-21] Gloor GB, Macklaim JM, Pawlowsky-Glahn V, Egozcue JJ (2017). Microbiome datasets are compositional: and this is not optional. Frontiers in Microbiology.

[ref-22] Gold Z, Shelton AO, Casendino HR, Duprey J, Gallego R, Van Cise A, Fisher M, Jensen AJ, D’Agnese E, Andruszkiewicz Allan E, Ramón-Laca A, Garber-Yonts M, Labare M, Parsons KM, Kelly RP (2023). Signal and noise in metabarcoding data. PLOS ONE.

[ref-23] Guri G, Shelton AO, Kelly RP, Yoccoz N, Johansen T, Præbel K, Hanebrekke T, Ray JL, Fall J, Westgaard J-I (2024). Predicting trawl catches using environmental DNA. ICES Journal of Marine Science.

[ref-24] Hickey BM, Banas NS (2003). Oceanography of the U.S. Pacific Northwest Coastal Ocean and estuaries with application to coastal ecology. Estuaries.

[ref-25] Hickey B, Geier S, Kachel N, MacFadyen A (2005). A bi-directional river plume: the Columbia in summer. Continental Shelf Research.

[ref-26] Hickey BM, Kudela RM, Nash JD, Bruland KW, Peterson WT, MacCready P, Lessard EJ, Jay DA, Banas NS, Baptista AM, Dever EP, Kosro PM, Kilcher LK, Horner-Devine AR, Zaron ED, McCabe RM, Peterson JO, Orton PM, Pan J, Lohan MC (2010). River influences on shelf ecosystems: introduction and synthesis. Journal of Geophysical Research: Oceans.

[ref-27] Hoshino T, Nakao R, Doi H, Minamoto T (2021). Simultaneous absolute quantification and sequencing of fish environmental DNA in a mesocosm by quantitative sequencing technique. Scientific Reports.

[ref-28] Hunter JR, Butler JL, Kimbrell C, Lynn EA (1990). Bathymetric patterns in size, age, sexual maturity, water content, and caloric density of Dover sole, *Microstomus pacificus*. CalCOFI Reports.

[ref-29] Jacobsen MW, Nygaard R, Hansen BK, Broberg M, Hansen MM, Hedeholm R, Nielsen EE (2023). Observing the Arctic: a comparison of environmental DNA (eDNA) and electrofishing for monitoring Arctic char and Atlantic salmon. Environmental DNA.

[ref-30] Jeunen GJ, Knapp M, Spencer HG, Lamare MD, Taylor HR, Stat M, Bunce M, Gemmell NJ (2019). Environmental DNA (eDNA) metabarcoding reveals strong discrimination among diverse marine habitats connected by water movement. Molecular Ecology Resources.

[ref-31] Jo T, Murakami H, Masuda R, Sakata MK, Yamamoto S, Minamoto T (2017). Rapid degradation of longer DNA fragments enables the improved estimation of distribution and biomass using environmental DNA. Molecular Ecology Resources.

[ref-32] Johnson K, Edwards A, Berger A, Grandin C, Wetzel C (2025). Status of the Pacific Hake (whiting) stock in U.S. and Canadian waters in 2025.

[ref-33] Kaplan I, Francis T, Punt A, Koehn L, Curchitser E, Hurtado-Ferro F, Johnson K, Lluch-Cota S, Sydeman W, Essington T, Taylor N, Holsman K, MacCall A, Levin P (2019). A multi-model approach to understanding the role of Pacific sardine in the California current food web. Marine Ecology Progress Series.

[ref-34] Karlsson E, Ogonowski M, Sundblad G, Sundin J, Svensson O, Nousiainen I, Vasemägi A (2022). Strong positive relationships between eDNA concentrations and biomass in juvenile and adult pike (*Esox lucius*) under controlled conditions: implications for monitoring. Environmental DNA.

[ref-35] Kawaguchi K, Moser HG (1993). Development and distribution of the early life history stages of the mesopelagic fish *Tactostoma macropus* (Stomiidae) in the transitional waters of the North Pacific.

[ref-36] Keller AA, Wallace JR, Methot RD (2017). The Northwest fisheries science center’s West Coast groundfish bottom trawl survey: history, design, and description.

[ref-37] Kim S, Barth JA (2011). Connectivity and larval dispersal along the Oregon coast estimated by numerical simulations. Journal of Geophysical Research.

[ref-38] Kirtane A, Wieczorek D, Noji T, Baskin L, Ober C, Plosica R, Chenoweth A, Lynch K, Sassoubre L (2021). Quantification of environmental DNA (eDNA) shedding and decay rates for three commercially harvested fish species and comparison between eDNA detection and trawl catches. Environmental DNA.

[ref-39] Laidig TE, Chess JR, Howard DF (2007). Relationship between abundance of juvenile rockfishes (*Sebastes* spp.) and environmental variables documented off northern California and potential mechanisms for the covariation. Fisheries Bulletin.

[ref-40] Langlois VS, Allison MJ, Bergman LC, To TA, Helbing CC (2021). The need for robust qPCR-based eDNA detection assays in environmental monitoring and species inventories. Environmental DNA.

[ref-41] Laschever E, Kelly R, Hoge M, Lee K (2023). Next generation of environmental monitoring: environmental DNA in agency practice. Columbia Journal of Environmental Law.

[ref-42] Ledger KJ, Everett M, Nichols KM, Larson WA, Baetscher DS (2025). Development and validation of an eDNA primer set for rockfishes in the Alaska current provides a new tool for fisheries management. Environmental DNA.

[ref-43] Ledger KJ, Hicks MBR, Hurst TP, Larson W, Baetscher DS (2024). Validation of environmental DNA for estimating proportional and absolute biomass. Environmental DNA.

[ref-44] Levi T, Allen JM, Bell D, Joyce J, Russell JR, Tallmon DA, Vulstek SC, Yang C, Yu DW (2019). Environmental DNA for the enumeration and management of Pacific salmon. Molecular Ecology Resources.

[ref-45] Litz MNC, Heppell SA, Emmett RL, Brodeur RD (2008). Ecology and distribution of the northern subpopulation of northern anchovy (Engraulis mordax) off the U.S. West Coast. CalCOFI Reports.

[ref-46] Mächler E, Osathanunkul M, Altermatt F (2018). Shedding light on eDNA: neither natural levels of UV radiation nor the presence of a filter feeder affect eDNA-based detection of aquatic organisms. PLOS ONE.

[ref-47] Maes SM, Desmet S, Brys R, Sys K, Ruttink T, Maes S, Hostens K, Vansteenbrugge L, Derycke S (2023). Detection and quantification of two commercial flatfishes *Solea solea* and *Pleuronectes platessa* in the North Sea using environmental DNA. Environmental DNA.

[ref-48] Martin M (2011). Cutadapt removes adapter sequences from high-throughput sequencing reads. EMBnet.journal.

[ref-49] McLaren MR, Willis AD, Callahan BJ (2019). Consistent and correctable bias in metagenomic sequencing experiments. eLife.

[ref-50] Miya M, Sato Y, Fukunaga T, Sado T, Poulsen JY, Sato K, Minamoto T, Yamamoto S, Yamanaka H, Araki H, Kondoh M, Iwasaki W (2015). MiFish, a set of universal PCR primers for metabarcoding environmental DNA from fishes: detection of more than 230 subtropical marine species. Royal Society Open Science.

[ref-51] Moku M, Kawaguchi K, Watanabe H, Ohno A (2000). Feeding habits of three dominant myctophid fishes, Diaphus theta, Stenobrachius leucopsarus and S. nannochir, in the subarctic and transitional waters of the western North Pacific. Marine Ecology Progress Series.

[ref-52] Muenzel D, Bani A, De Brauwer M, Stewart E, Djakiman C, Halwi, Purnama R, Yusuf S, Santoso P, Hukom FD, Struebig M, Jompa J, Limmon G, Dumbrell A, Beger M (2024). Combining environmental DNA and visual surveys can inform conservation planning for coral reefs. Proceedings of the National Academy of Sciences of the United States of America.

[ref-53] Nevers MB, Byappanahalli MN, Morris CC, Shively D, Przybyla-Kelly K, Spoljaric AM, Dickey J, Roseman EF (2018). Environmental DNA (eDNA): a tool for quantifying the abundant but elusive round goby (*Neogobius melanostomus*). PLOS ONE.

[ref-54] Ono K, Shelton AO, Ward EJ, Thorson JT, Feist BE, Hilborn R (2016). Space-time investigation of the effects of fishing on fish populations. Ecological Applications.

[ref-55] Parnel MM, Emmett RL, Brodeur RD (2008). Ichthyoplankton community in the Columbia River plume off Oregon: effects of fluctuating oceanographic conditions. Fishery Bulletin.

[ref-56] Pont D (2024). Predicting downstream transport distance of fish eDNA in lotic environments. Molecular Ecology Resources.

[ref-57] Quigley L, Franks P, Thompson A, Field J, Santora J (2024). Quantifying the fates and retention of larval rockfish through Lagrangian analyses. Marine Ecology Progress Series.

[ref-58] Ramón-Laca A, Wells A, Park L (2021). A workflow for the relative quantification of multiple fish species from oceanic water samples using environmental DNA (eDNA) to support large-scale fishery surveys. PLOS ONE.

[ref-59] R Core Team (2024). R: a language and environment for statistical computing.

[ref-60] Reynolds JA (2001). Habitat associations and distribution of widow rockfish, Sebastes entomelas, with implications for marine reserve design.

[ref-91] Rourke ML, Fowler AM, Hughes JM, Broadhurst MK, DiBattista JD, Fielder S, Wilkes Walburn J, Furlan EM (2022). Environmental DNA (eDNA) as a tool for assessing fish biomass: a review of approaches and future considerations for resource surveys. Environmental DNA.

[ref-61] Ruppert KM, Davis DR, Rahman MS, Kline RJ (2022). Development and assessment of an environmental DNA (eDNA) assay for a cryptic Siren (Amphibia: Sirenidae). Environmental Advances.

[ref-62] Salter I, Joensen M, Kristiansen R, Steingrund P, Vestergaard P (2019). Environmental DNA concentrations are correlated with regional biomass of Atlantic cod in oceanic waters. Communications Biology.

[ref-63] Sato M, Barth J, Benoit-Bird K, Pierce S, Cowles T, Brodeur R, Peterson W (2018). Coastal upwelling fronts as a boundary for planktivorous fish distributions. Marine Ecology Progress Series.

[ref-64] Shaffer MR, Allan EA, Kelly RP, Adams NG, Moore SK, Brasseale E, Brandão-Dias PFP, Scott OM, Shelton AO, Parsons KM (2026). Non-target detections change the interpretation of environmental DNA communities. Metabarcoding and Metagenomics.

[ref-65] Shaffer MR, Andruszkiewicz Allan E, Van Cise AM, Parsons KM, Shelton AO, Kelly RP (2025). Observation bias in metabarcoding. Molecular Ecology Resources.

[ref-66] Shelton AO, Gold ZJ, Jensen AJ, D′Agnese E, Andruszkiewicz Allan E, Van Cise A, Gallego R, Ramón-Laca A, Garber-Yonts M, Parsons K, Kelly RP (2023). Toward quantitative metabarcoding. Ecology.

[ref-67] Shelton AO, Ramón-Laca A, Wells A, Clemons J, Chu D, Feist BE, Kelly RP, Parker-Stetter SL, Thomas R, Nichols KM, Park L (2022). Environmental DNA provides quantitative estimates of Pacific hake abundance and distribution in the open ocean. Proceedings of the Royal Society B: Biological Sciences.

[ref-68] Smith KF, Brown JH (2002). Patterns of diversity, depth range and body size among pelagic fishes along a gradient of depth. Global Ecology and Biogeography.

[ref-69] Somers KA, Pfeiffer L, Miller S, Morrison W (2018). Using incentives to reduce bycatch and discarding: results under the West Coast catch share program. Coastal Management.

[ref-70] Stan Development Team (2023). Stan modeling language users guide and reference manual. https://mc-stan.org/.

[ref-71] Steinbeck J (1945). Cannery row.

[ref-72] Stoeckle MY, Ausubel JH, Coogan M (2022). 12S gene metabarcoding with DNA standard quantifies marine bony fish environmental DNA, identifies threshold for reproducible detection, and overcomes distortion due to amplification of non-fish DNA. Environmental DNA.

[ref-73] Strickler KM, Fremier AK, Goldberg CS (2015). Quantifying effects of UV-B, temperature, and pH on eDNA degradation in aquatic microcosms. Biological Conservation.

[ref-74] Suntsov A, Brodeur R (2008). Trophic ecology of three dominant myctophid species in the northern California current region. Marine Ecology Progress Series.

[ref-75] Takahara T, Minamoto T, Yamanaka H, Doi H, Kawabata Z (2012). Estimation of fish biomass using environmental DNA. PLOS ONE.

[ref-76] Tissot BN, Wakefield WW, Hixon MA, Clemons J (2008). Twenty years of fish-habitat studies on Heceta Bank, Oregon.

[ref-77] Tolimieri N, Shelton AO, Feist BE, Simon V (2015). Can we increase our confidence about the locations of biodiversity ‘hotspots’ by using multiple diversity indices?. Ecosphere.

[ref-78] Ushio M, Murakami H, Masuda R, Sado T, Miya M, Sakurai S, Yamanaka H, Minamoto T, Kondoh M (2018). Quantitative monitoring of multispecies fish environmental DNA using high-throughput sequencing. Metabarcoding and Metagenomics.

[ref-79] Veilleux HD, Misutka MD, Glover CN (2021). Environmental DNA and environmental RNA: current and prospective applications for biological monitoring. Science of the Total Environment.

[ref-80] Vetter RD, Lynn EA, Garza M, Costa AS (1994). Depth zonation and metabolic adaptation in Dover sole, *Microstomus pacificus*, and other deep-living flatfishes: factors that affect the sole. Marine Biology.

[ref-81] Willis JM, Pearcy WG (1982). Vertical distribution and migration of fishes of the lower mesopelagic zone off Oregon. Marine Biology.

[ref-82] Xiong J, MacCready P, Brasseale E, Andruszkiewicz Allan E, Ramón-Laca A, Parsons KM, Shaffer M, Kelly RP (2025). Advective transport drives environmental DNA dispersal in an estuary. Environmental Science & Technology.

[ref-83] Yamamoto S, Minami K, Fukaya K, Takahashi K, Sawada H, Murakami H, Tsuji S, Hashizume H, Kubonaga S, Horiuchi T, Hongo M, Nishida J, Okugawa Y, Fujiwara A, Fukuda M, Hidaka S, Suzuki KW, Miya M, Araki H, Yamanaka H, Maruyama A, Miyashita K, Masuda R, Minamoto T, Kondoh M (2016). Environmental DNA as a ‘Snapshot’ of fish distribution: a case study of Japanese Jack Mackerel in Maizuru Bay, Sea of Japan. PLOS ONE.

[ref-84] Zhang S, Lu Q, Wang Y, Wang X, Zhao J, Yao M (2020). Assessment of fish communities using environmental DNA: effect of spatial sampling design in lentic systems of different sizes. Molecular Ecology Resources.

[ref-85] Zwolinski JP, Demer DA (2024). An updated model of potential habitat for northern stock Pacific Sardine (*Sardinops sagax*) and its use for attributing survey observations and fishery landings. Fisheries Oceanography.

