# Supplementary material for: Quantitative eDNA abundances for multispecies monitoring along the US West Coast
Source: PeerJ. 2026 Nov 9;14:e21810. doi: 10.7717/peerj.21810 (PMC13757736; doi:10.7717/peerj.21810)
Supplement: Supplementary file 1 [file peerj-14-21810-s002.pdf]

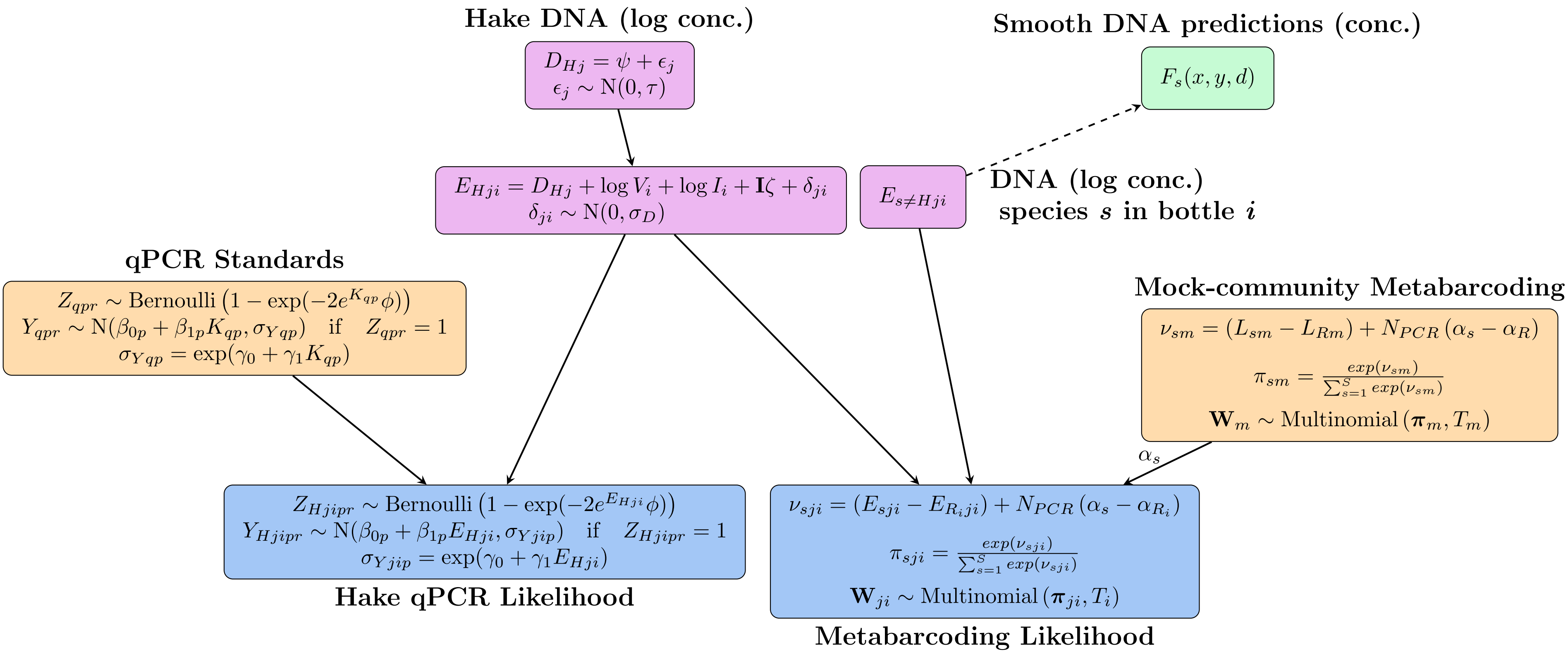

Index

s = species (H = Hake)
j = location (northing, easting, depth)
x = northing
y = easting
d = depth
i = biological replicate
r = technical replicate
p = qPCR plate
m = mock community sample
q = qPCR standard aliquote sample
R = metabarcoding reference species

Parameters and derived quantities

ψ = mean hake eDNA concentration (log(copies/μL)
δ = biological replicate random effect
V_i = volume filtered and aliquote dilution offset
ϕ = qPCR amplification parameter
σ_Y = standard deviation of qPCR Ct values
β_{0p}, β_{1p} = intercept and slope defining mean qPCR - DNA concentration relationship
γ_0, γ_1 = intercept and slope defining standard deviation of qPCR - DNA concentration relationship
α_s = amplification efficiency relative to reference species
π = predicted sequence proportions at the end of PCR
ζ = ethanol wash effect
τ = standard deviation among biological replicates

Observations

Z = qPCR amplification (yes=1; no=0)
Y = qPCR Ct values
K = known DNA conc. (log(copies/μL))
L = known DNA conc. (log(copies/μL))
W = metabarcoding sequencing reads
T = total number sequencing reads
V = volume filtered and aliquote dilution offset
I = aliquote dilution factor

States

D = DNA conc. at a station-depth (log(copies/μL))
E = DNA conc. in a biological replicate (log(copies/μL))
F = Smoothed DNA conc. (copies/L)
