# Supplementary material for: Quantitative eDNA abundances for multispecies monitoring along the US West Coast
Source: PeerJ. 2026 Nov 9;14:e21810. doi: 10.7717/peerj.21810 (PMC13757736; doi:10.7717/peerj.21810)
Supplement: Supplementary file 3 [file peerj-14-21810-s006.docx]

**Supplemental Table S1. Initial mock community proportional abundances.**

The initial proportional abundances were measured by amplifying the 12S rRNA gene using MarVer1 primers (Valsecchi et al., 2020) and ddPCR QX200 Droplet Digital PCR system (Bio-Rad, Inc.)) of the selected species (n=12) across different mock communities (n=8) each containing three replicates (total of 24 samples). NA represent no DNA template included.

| Species | Mock1 | | Mock2 | | Mock3 | | Mock4 | |
| --- | --- | --- | --- | --- | --- | --- | --- | --- |
|  | Even | Skew | Even | Skew | Even | Skew | Even | Skew |
| *Clupea pallasii* | 0.437 | 0.325 | 0.042 | 0.009 | - | - | 0.025 | 0.038 |
| *Engraulis mordax* | 0.122 | 0.034 | 0.15 | 0.15 | 0.146 | 0.16 | 0.308 | 0.478 |
| *Leuroglossus stilbius* | 0.108 | 0.29 | - | - | - | - | - | - |
| *Merluccius productus* | 0.079 | 0.144 | 0.12 | 0.06 | 0.117 | 0.075 | 0.247 | 0.287 |
| *Microstomus pacificus* | - | - | 0.057 | 0.215 | - | - | - | - |
| *Sardinops sagax* | 0.028 | 0.033 | 0.14 | 0.035 | 0.137 | 0.025 | 0.289 | 0.131 |
| *Scomber japonicus* | - | - | 0.122 | 0.053 | 0.119 | 0.027 | - | - |
| *Sebastes entomelas* | - | - | - | - | 0.068 | 0.218 | - | - |
| *Stenobrachius leucopsarus* | - | - | - | - | 0.356 | 0.487 | - | - |
| *Tactostoma macropus* | - | - | 0.13 | 0.324 | - | - | - | - |
| *Tarletonbeania crenularis* | - | - | 0.083 | 0.124 | - | - | - | - |
| *Thaleichthys pacificus* | 0.123 | 0.113 | 0.099 | 0.012 | - | - | 0.013 | 0.021 |
| *Trachurus symmetricus* | 0.103 | 0.06 | 0.057 | 0.018 | 0.056 | 0.008 | 0.118 | 0.04 |
