# Supplementary material for: Quantitative eDNA abundances for multispecies monitoring along the US West Coast
Source: PeerJ. 2026 Nov 9;14:e21810. doi: 10.7717/peerj.21810 (PMC13757736; doi:10.7717/peerj.21810)
Supplement: Supplementary file 4 [file peerj-14-21810-s007.docx]

Figure S4. Estimated species (Clupea pallasii,	Engraulis mordax, and Leuroglossus stilbius) eDNA concentration (copies/L; first row) through the joint model and their respective standard deviation distribution (SD) of the of log-transformed confidence intervals (second row) across 0, 50, 150, 300, and 500m depth samples. Spatially smoothed eDNA concentrations for the same species and depths are shown in the third row, with the associated standard error (SE) of prediction from the spatial smoothing model (fourth row).

Figure S4 (continued). Species Microstomus pacificus, Sardinops sagax, and Scomber japonicus.

Figure S4 (continued). Species Sebastes entomelas, Stenobrachius leucopsarus, and Tactostoma macropus.

Figure S4 (continued). Species Tarletonbeania crenularis, Thaleichthys pacificus, and Trachurus symmetricus.
